# Effects of non-pharmacological interventions on the depressive outcomes in people with mild cognitive impairment: an overview of systematic reviews

**DOI:** 10.3389/fpsyt.2024.1415113

**Published:** 2025-01-15

**Authors:** Chen Yaxin, Yan Lijiao, Chen Zhao, Hu Ziteng, Zhang Fuqiang, Liu Zhenhong, Feng Luda, Li Yixiang, Dai Xiangwei, Che Qianzi, Li Huizhen, Zhang Haili, Liang Ning, Shi Nannan

**Affiliations:** ^1^ Institute of Basic Research in Clinical Medicine, China Academy of Chinese Medical Sciences, Beijing, China; ^2^ Department of Epidemiology and Data Science, Amsterdam University Medical Centers, University of Amsterdam, Amsterdam, Netherlands; ^3^ Institute for Brain Disorders, Dongzhimen Hospital, Beijing University of Chinese Medicine, Beijing, China; ^4^ Dongfang Hospital, Beijing University of Chinese Medicine, Beijing, China

**Keywords:** mild cognitive impairment, depression, non-pharmacological interventions, music therapy, exercise therapy, cognitive interventions, psychosocial intervention, health education

## Abstract

**Objective:**

This study aimed to summarize and assess the certainty of evidence of non-pharmacological interventions (NPIs) on the depressive outcomes in people with mild cognitive impairment (MCI) based on published systematic reviews (SRs).

**Method:**

Databases including PubMed, EMBASE, PsycINFO, the Cochrane Database of Systematic Reviews, CNKI, CBM, Wanfang and VIP database were searched from their inception to June 6, 2023. The methodological quality of the SRs was evaluated using the AMSTAR2 tool, and the quality of evidence was assessed using the Grading of Recommendation Assessment, Development and Evaluation (GRADE) framework.

**Results:**

Twelve eligible SRs were included. Three SRs focused on cognitive interventions (general, computer-based, cognitive stimulation/rehabilitation), six reviews on physical activity (Tai Chi, exercise therapy, dance), three on psychosocial interventions including cognitive behavioral therapy (CBT), mindfulness-based intervention (MBI) and type not specified, one on music therapy, and one on health education; moreover, there were two SRs on multimodal NPIs. One Cochrane SR was rated as moderate quality, while the others were rated as low quality according to AMSTAR2. The overlap between primary studies of included SRs (a total of 51 studies) was 1.8%, indicating slight overlap. General cognitive interventions (SMD=-0.25, 95% CI [−0.46, −0.04], GRADE: moderate) and computer-based cognitive interventions (narrative evidence) showed potential benefits in improving depression. Exercise therapy showed consistency between two SRs in benefiting depressive symptoms of MCI (SMD=-0.33, 95% CI [−0.56, −0.10], GRADE: Low; SMD=−0.37, 95% CI [-0.64, -0.10], GRADE: Low). Dance (SMD=−0.37, 95% CI [-1.11, 0.38], GRADE: Low), CBT (SMD=0.03,95% CI [-0.18, 0.24], GRADE: Moderate), MBI (SMD=0.29, 95% CI [0.00, 0.57], GRADE: Very Low) and health education (SMD=-0.12, 95% CI [−0.44, 0.20], GRADE: Low) did not show significant difference compared to control group in improving depressive symptoms, while the effectiveness of Tai Chi, music therapy and multimodal NPIs showed inconsistency across different studies.

**Conclusion:**

Cognitive interventions (general or computer-based) and exercise therapy (a type of physical activity) show preliminary potential to improve depressive symptoms, while others do not show significant effects or relate to confused effects. Further methodologically rigorous and adequately powered primary studies are necessary for each of these NPIs, with reporting on the components of the interventions clearly in MCI patients.

## 1 Introduction

Mild cognitive impairment (MCI) represents a critical transitional phase between healthy aging and dementia, affecting approximately 15.56% of individuals aged 50 years and older globally (1, 2). Depression, a common comorbidity in MCI, complicates the condition, affecting up to 32% of those with MCI (3–5). The presence of depression in MCI patients is particularly concerning, as it significantly heightens the risk of progression to dementia. A comprehensive meta-analysis of 32 studies involving over 62,000 participants suggests that depression nearly doubles the risk of developing dementia (6). This review also noted a trend toward smaller effect sizes in studies with longer follow-ups, indicating that depression may play a prodromal role in dementia. Therefore, effective management strategies for depression in MCI are crucial, underscoring the need for interventions aimed at alleviating depressive symptoms to potentially slow the progression toward dementia.

While antidepressants are a standard treatment for depression, their effectiveness in preventing cognitive decline and dementia in MCI is not well-established. The World Health Organization (WHO) has highlighted the lack of sufficient evidence to support the use of antidepressants for reducing the risk of cognitive decline and/or dementia in this population (7). Given these challenges, non-pharmacological interventions recommended by the WHO emerge as important alternatives (7).

Currently, various non-pharmacological interventions, such as psychological therapies (e.g., cognitive behavioral therapy, problem-solving therapy, interpersonal therapy, and behavioral activation), are used to improve depressive symptoms in individuals with MCI. However, their effectiveness remains uncertain. Although several systematic reviews have assessed these interventions, their findings are inconsistent. For instance, one review suggests that cognitive interventions are effective in alleviating depressive symptoms (8), while another one by Xu et al. reports them as ineffective (9). These discrepancies may result from methodological differences, sample characteristics, or varying applicability of interventions across patient populations. Such conflicting results contribute to the challenges faced by patients and physicians in selecting appropriate treatments.

Therefore, a comprehensive overview of systematic reviews is needed to integrate and compare findings, providing a more reliable evidence base to guide clinical practice and decision-making. Our study aims to fill this gap by conducting an overview of systematic reviews on non-pharmacological interventions for depression in MCI. By doing so, it is expected to provide a clear evidence-based understanding of these non-pharmacological interventions’ effectiveness, aiding in the informed management of depression in MCI and potentially mitigating progression to dementia.

## 2 Methods

### 2.1 Eligibility criteria

#### 2.1.1 Type of studies

Systematic reviews and meta-analyses were included, with no language limitation and no restrictions on the study design of primary studies. Conference abstracts were excluded.

#### 2.1.2 Population

Patients included in the systematic reviews should have been diagnosed with MCI using Petersen criteria (10), the National Institute on Aging and Alzheimer’s Association (NIA-AA) criteria (11); or clear diagnostic criteria based on neuropsychological testing was also accepted if well described. Patients diagnosed with dementia were excluded. If both MCI and dementia patients were involved, only systematic reviews with separate analyses on MCI patients were included.

#### 2.1.3 Interventions

Non-pharmacological intervention is any type of healthcare intervention which is not primarily based on medications. It included, but not limited to, physical activity, cognitive interventions, and music therapy. The intervention groups did not include pharmacological therapies, such as antidepressants, unless such therapies were administered equally to both the intervention and control groups.

#### 2.1.4 Comparison

The comparison focused on placebo, no treatment, treatment as usual, and pharmacological therapies.

#### 2.1.5 Outcomes

The primary outcome was the change in depression or depressive symptoms assessed using scales such as the Beck Depression Inventory (BDI), Cornell Depression Score (CDS), Cornell Scale for Depression in Dementia (CSDD), Geriatric Depression Scale (GDS), Hospital Anxiety and Depression Scale (HADS), Montgomery-Asberg Depression Scale (MADRS), and Patient Health Questionnaire-9 (PHQ-9).

### 2.2 Literature search

Electronic databases including PubMed, EMBASE, PsycINFO, Cochrane Database of Systematic Reviews, CNKI, CBM, Wanfang and the VIP database were systematically searched from inception to June 6, 2023. The detailed search strategy is provided in the **Supplementary Table 1**.

### 2.3 Study selection and data collection

After excluding duplicate publications, two authors screened the titles and abstracts, followed by the full texts, independently for study eligibility based on the inclusion criteria. Disagreements were resolved through discussion or consultation with a third author.

Two authors independently extracted the data of included systematic reviews using a specifically designed extraction form. The details of author, date of review, intervention and comparator, number of participants, diagnosis criteria, outcomes examined, meta-analysis or narrative results were extracted. All discrepancies were resolved through discussion or consultation with a third author.

### 2.4 Assessment of methodological quality of included reviews

The methodological quality of eligible reviews was appraised by two authors independently using AMSTAR2 tool (12). Disagreements were resolved through discussion or consultation with a third author.

AMSTAR2 includes 16 items with 7 critical items (protocol registered before commencement of the review, adequacy of the literature search, justification for excluding individual studies, risk of bias from individual studies being included in the review, appropriateness of meta-analytical methods, consideration of risk of bias when interpreting the results of the review, assessment of presence and likely impact of publication bias) (12). Items will be recorded as ‘Yes’, ‘No’, ‘Partial Yes’, ‘Includes only NRSI’, ‘Includes only RCTs’ or ‘No meta-analysis conducted’. Reviews with no or one non-critical weakness are rated as high quality. Reviews with more than one non-critical weakness are rated as moderate quality. Reviews with one critical flaw with or without non-critical weaknesses are rated as low quality.

### 2.5 Assessment of certainty of evidence

Two authors independently appraised the certainty of evidence from meta-analyses using the GRADE approach. Any disagreement was resolved by discussion or consultation with a third author.

In the GRADE system, the certainty of evidence is classified as: high, moderate, low, or very low. Evidence based on randomized controlled trials begins as high-quality. Five reasons (study limitation, imprecision, inconsistency of result, indirectness of evidence and reporting bias) may decrease confidence in the evidence (13).

### 2.6 Data synthesis

A narrative analysis was conducted to summarize the results from the included reviews. Meta-analysis was not performed due to the heterogeneity in study designs among the primary studies. Corrected covered area (CCA) index was assessed to quantify the degree of overlap between systematic reviews to be pooled in an overview (14). CCA values were categorized as representing slight (0-5%), moderate (6-10%), high (11-15%), or very high (>15%) overlap (15). CCA was calculated for the total number of included systematic reviews and for individual non-pharmacological interventions with more than two systematic reviews. Subgroup analyses will be performed regarding amnestic versus non-amnestic MCI, MCI due to Alzheimer’s disease versus due to other conditions when data available for the purpose. The effects of various non-pharmacological interventions were presented in forest plots using Python version 3.15 (Python Software Foundation) and the Matplotlib library (16). The plots were based on the meta-analysis results of the included studies.

## 3 Results

### 3.1 Search results

**Figure 1:**
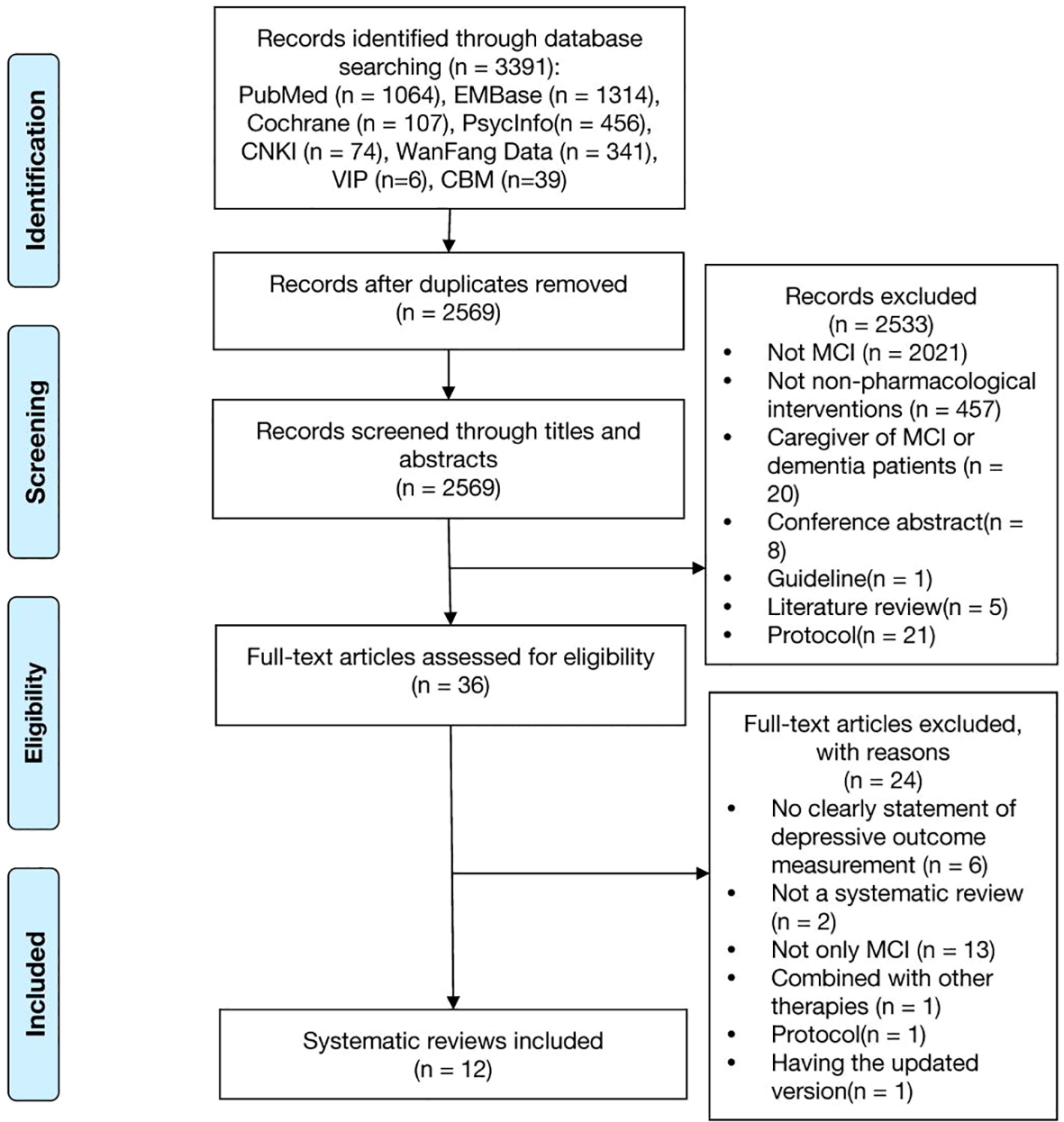
Flow diagram of the systematic review identification process. A lot of studies were excluded during the title and abstract screening because the keywords we used in search strategy (**Supplementary Table 1**) were not specific, such as, cognitive dysfunction, cognitive disorder, neurocognitive disorder, cognitive decline, mental deterioration, which lead to excluding many studies were not specific on MCI. And we also didn’t set any keywords on non-pharmacological interventions to obtain as much study as possible, which also lead to excluding many studies were not specific on non-pharmacological interventions. In **Supplementary Table 2** there were all 24 out of 36 papers excluded after full-text reading, leaving 12 papers included.

A total of 3,391 reviews were identified. After excluding duplicates, 2569 references were screened through titles and abstracts, of these 36 full-texts were read. Totally, 12 systematic reviews were included. The detailed selection process is illustrated in **Figure 1**.

### 3.2 Study characteristics

Among the 12 systematic reviews (9, 17–27), 9 reviews included only RCTs (18, 20–27), one review included pre-test and post-test data from both the intervention and control groups (19), one included RCTs or a cross-over design (9), and one included RCTs or observational studies (17). Only one review focused on a specific subtype, amnestic MCI (24), while the others investigated MCI without specifying the subtypes. Some studies, such as Zhang et al. (26) and Li et al. (20), explicitly used Petersen criteria or other established definitions of MCI. However, several studies, including Li et al. (19)and Wu et al. (25), did not specify the exact diagnostic criteria employed. Three reviews focused on cognitive interventions (9, 19, 24), six reviews on physical activities (9, 20, 21, 25–27), three on psychosocial interventions (9, 18, 23), one on music therapy (17), one on health education (9); moreover, there were two on multimodal non-pharmacological interventions (9, 22). None of the included systematic reviews required the presence of depressive symptoms as an inclusion criterion; instead, depressive symptoms were evaluated as an outcome. Most reviews (10/12) used GDS as the measurement index of depression (9, 17–21, 23–25, 27), while three reviews used BDI (17, 19, 22), 2 used CDS (26, 27), 2 used HADS (19, 25), 1 used Goldberg scale (19), 1 used PHQ-9 (23), and 1 used CSDD (21). The characteristics of included reviews are detailed in **Table 1**.

**Table 1:** Basic characteristics of included systematic reviews.

Study ID	No. studies No. participants	Study design included	Population			Intervention	Control	Depression scales	Outcome	AMSTAR2
Li 2011 (19)	Intervention group: 6(135); Control group: 3 (39)	Intervention study with pre-test and post-test data, with or without control group	MCI	No clear diagnosis criteria	NR	Cognitive intervention (cognitive stimulation training or cognitive rehabilitation)	Placebo, no treatment, waiting list;	Self-rated depression (BDI; GDS; HADS; Goldberg scale)	Post-test/pre-test data:Weighted effect size MCI intervention group: 0.35(0.11 to 0.60) MCI control group: 0.28(−0.17 to 0.72)	Critically low
Simon 2012 (24)	1(39)	RCT	aMCI	Petersen’s criteria for A-MCI (e.g. Petersen al., 2001)	NR	Cognitive intervention (cognitive computer-based training) and cholinesterase inhibitors	Cholinesterase inhibitors	GDS	No data	Critically low
Mei 2019 (22)	2(52)	RCT	MCI	Patients meeting various MCI diagnostic criteria, but must fulfill the following: cognitive decline without reaching the criteria for dementia, and overall daily living abilities remaining normal. Excluded are studies involving a mix of MCI patients, dementia patients, and healthy older adults; also excluded are patients with cognitive impairment caused by secondary reasons	NR	Multimodal non-pharmacological interventions	Placebo or active control	MADRS; BDI	SMD=-0.83, 95%CI (-1.41, -0.26)	Critically low
Zhang 2019 (26)	2(594)	RCT	MCI	Individuals with MCI were identified by any available diagnostic criteria, such as Petersen criteria, Mayo clinic criteria for amnestic MCI and other standards and consensus. RCTs including participants with MCI for whom the cognitive deficits may be due to medical or neurological disorders (e.g., Alzheimer’s disease, dementia and Parkinson’s disease)	older adults (≥60 years)	Physical activity (Tai Chi alone without other treatments).	No treatment, TAU or other exercises that are different from Tai Chi	CDS	MD=0.02, 95% CI=- 0.23 to 0.27	Critically low
Zhang 2020 (27)	4(730)	RCT	MCI	Meeting Petersen criteria or diagnosed with MCI according to the MMSE	older adults (≥60 years)	Physical activity (Tai Chi: only Tai Chi exercise interventions were accepted, with no restrictions on the form, frequency, duration, or location of the exercise)	TAU, other forms of exercise, or no treatment	GDS; CDS	SMD = 0.00, 95% CI(-0.14,0.15) Subgroup: GDS: SMD = -0.02 (-0.36 to 0.31) CDS: SMD = 0.01 (-0.15 to 0.18)	Critically low
Li 2021 (20)	2(110)	RCT	MCI	Based on the diagnostic criteria for MCI proposed by the Petersen team and the standards for MCI outlined in the MMSE and MoCA.	NR	Physical activity (Tai Chi: the systematic and regular Tai Chi training can include forms such as Yang-style Tai Chi, Chen-style Tai Chi, Sun-style Tai Chi, and the simplified 24-form Tai Chi.)	TAU, or no treatment, health education, or stretching exercises are allowed, but specialized cognitive interventions and regular aerobic exercise are prohibited	GDS	MD = -2.81, 95%CI (-3.48, -2.14)	Critically low
Wu 2021 (25)	3(157)	RCT	MCI	Participants with MCI	older adults	Physical activity (Dance)	TAU, active control	GDS, HADS-Deutsche	SMD=−0.37, 95% CI:-1.11 to 0.38	Critically low
Xu 2021 (9)	15(662)	RCT or a cross-over design with at least the first-period outcomes available	MCI	MCI of any type	older adults	Cognitive intervention (cognition-based intervention)	Non-active control group such as no treatment or usual lifestyle	GDS-15; GDS-30	SMD=-0.25, 95% CI: −0.46, −0.04	Critically low
	8(393)					Physical activity (exercise therapy)			SMD=-0.33, 95% CI: −0.56, −0.10	
	6(216)					Health education			SMD=-0.12, 95% CI: −0.44, 0.20	
	6(277)					Psychosocial intervention (type not specified)			SMD=-0.13, 95% CI: −0.40, 0.14	
	2(173)					Multimodal non-pharmacological interventions (exercise therapy combined with cognition-based intervention)			SMD=-0.33, 95% CI: −0.68, 0.02	
Jordan 2022 (17)	5(NR)	RCT or intervention study (single group design)	MCI	Individuals living with a clinical diagnosis of MCI. Excluded studies if participants had received a clinical diagnosis of dementia (mild, moderate or severe dementia) or subjective cognitive impairment	NR	Music therapy (active music therapy)	Gymnastics control group/No treatment,	GDS; BDI	NO DATA (Study reported significant improvements in depression scores from baseline, while another study failed to find any significant change.)	Critically low
	1(100)				NR	Music therapy (passive music therapy)	No control group		NO DATA [One study explored the benefits of listening to music or listening to music with dance routines (no control group). Significant improvements were found from baseline on measures of depression (BDI)]	
	1(44)				NR	Music therapy (reminiscence music therapy)	No control group		NO DATA (Two studies used music as a means of reminiscence. There was a decline in depression and anxiety scores at 3- and 9-months post intervention for music reminiscence group and art group; however, these reductions were not statistically significant)	
	1(16)				NR	Music therapy (music with movement)	No control group		NO DATA (A single group design study explored the benefits of a music with movement intervention, significant improvements were found for depression)	
Orgeta 2022 (23)	3(339)	RCT (including cluster randomised trials)	MCI	People with a diagnosis of MCI. Any definition of MCI was acceptable as long as the criteria used were published and included evidence of objective cognitive impairment but no dementia.	NR	Psychosocial intervention (cognitive behavioral therapy: it included CBT, PST, BA or behavior management therapy)	TAU	GDS-15; PHQ-9	SMD 0.03, 95% CI -0.18 to 0.24	Moderate
Leow 2023 (18)	4(196)	RCT	MCI	Diagnosed with MCI using any existing definitions of MCI or research set criteria. Excluded studies if participants had MCI due to other conditions not limited to chemotherapy, cancer and Parkinson disease	older adults (≥60 years)	Psychosocial intervention (mindfulness-based intervention: it incorporates the various components of mindfulness to create different modes of therapies such as mindfulness-based stress reduction (MBSR), mindfulness-based cognitive therapy (MBCT) and other variations in the treatment of different conditions.)	Active control or usual care	GDS	SMD = 0.29, p= 0.05, 95%CI = [0.00, 0.57]	Critically low
Liu 2023 (21)	6(837)	RCT	MCI	Participants with MCI	NR	Physical activity (Exercise therapy: Chinese square dance, Taiji, structured limbs-exercise, dance, multicomponent)	Non-active control (no treatment, waitlist, relaxation, placebo), TAU or health education	GDS-15; CSDD; GDS	SMD = -0.37, 95%CI: -0.64 to -0.10	Critically low

RCT, Randomized controlled trial; BDI, Beck Depression Inventory; CDS, Cornell Depression Score; CSDD, Cornell Scale for Depression in Dementia; GDS, Geriatric Depression Scale; HADS, Hospital Anxiety and Depression Scale; MADRS, Montgomery-Asberg Depression Scale; PHQ-9, Patient Health Questionnaire-9; NR, not reported; TAU, treatment as usual; MCI, mild cognitive impairment; MMSE, Mini-Mental State Examination; MoCA, Montreal Cognitive Assessment.

Ten systematic reviews conducted meta-analyses, while two performed narrative analysis (17, 24). The overlap between primary studies (a total of 51 studies) was 1.8%, indicating slight overlap. The detailed results of overlap are presented in the **Supplementary Table 3**.

### 3.3 Quality assessment

One Cochrane systematic review was rated as moderate quality (23), while the other systematic reviews were rated as low quality according to AMSTAR2. The main reasons for the low quality were low scores on two critical items, item 2 and item 7. The certainty of evidence for 14 meta-analyses in 9 systematic reviews was assessed using GRADE with most rated as low or very low certainty; only two were graded as moderate certainty. The detailed results of AMSTAR and GRADE are presented in **Supplementary Tables 4**, **5**.

### 3.4 Outcomes

**Figure 2:**
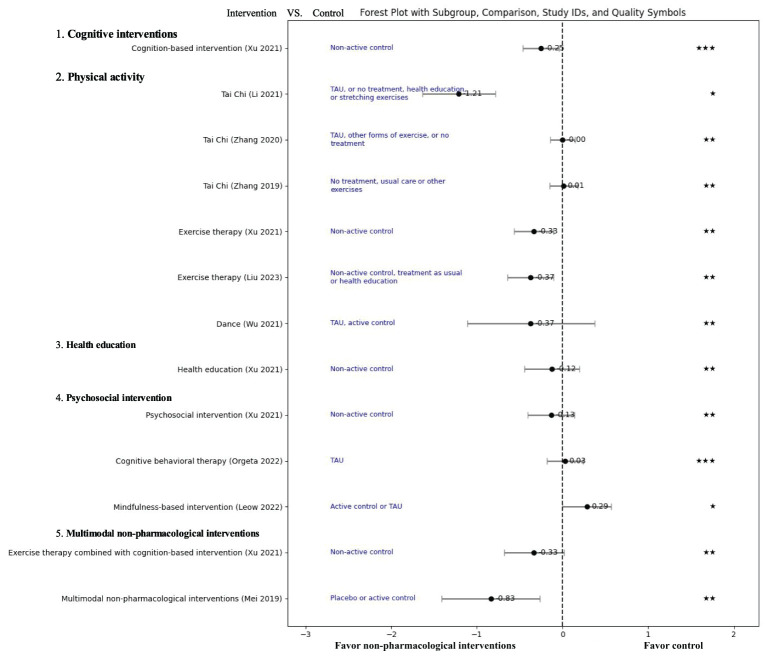
Effect of non-pharmacological treatments on the depressive outcomes in patients with mild cognitive impairment. TUA, Treatment as usual; SMD, Standardized mean difference; GRADE score: “High”: “★★★★”, “Moderate”: “★★★”, “Low”: “★★”, “Very Low”: “★”.

Results of the outcomes are presented in **Table 1** and **Figure 2**.

#### 3.4.1 Cognitive interventions

Cognitive intervention focuses on guided practice on tasks target at specific cognitive functions, such as memory, attention, or problem-solving, including but not limited to playing online games or completing cognitive tasks in alignment with a training regimen, playing video games that require visuospatial reasoning, and engaging in novel activities such as art, and music (28, 29). Three systematic reviews on cognitive interventions were included, covering a total of 24 primary studies, with an overlap of 2.1% (9, 19, 24).

Two systematic reviews favored cognitive intervention than the control group in improving depression for MCI patients. Xu et al. focused on cognition-based intervention, but not specifically clarified the content of the intervention (9). The analysis showed that cognitive intervention significantly improved depressive symptoms compared to a non-activity group (SMD=-0.25, 95% CI [−0.46, −0.04], 15 RCTs, 662 participants, GRADE: Moderate) (9). Simon et al. explored the cognitive intervention focusing on cognitive computer-based training (24). This review found that cognitive computer-based training combined with cholinesterase inhibitors was more effective than cholinesterase inhibitors alone in improving depression based on one RCT with 39 participants (24).

Li et al. focused on cognitive stimulation, training or rehabilitation, and conducted a meta-analysis using pre-test and post-test data, which found a small effect of cognitive intervention on self-rated depression, but the effect did not reach statistical significance between intervention (weighted effect size =0.35, 95% CI [0.11, 0.60], 6 studies, 135 participants) and control groups (weighted effect size =0.28, 95% CI [-0.17, 0.72], 3 studies, 39 participants) (Q=0.08, P=0.78) (19).

#### 3.4.2 Physical activity

Physical activity refers to any bodily movement produced by skeletal muscles that requires energy expenditure, including Tai Chi, exercise therapy and dance (7). Six systematic reviews assessed PA (9, 20, 21, 25–27), of which three were about Tai Chi (20, 26, 27), two about exercise therapy (9, 21) and one about dance (25).

##### 3.4.2.1 Tai Chi

Tai Chi is a traditional fitness art from China. Two systematic reviews found no difference between Tai Chi group and control group (MD=0.02, 95% CI [-0.23, 0.27], *P*=0.89, *I* ^2^ = 0%, 2 RCTs, 594 participants, GRADE: Low; SMD=0,00 95% CI [-0.14, 0.15], *P*=0.95, *I* ^2^ = 0%, 4 RCTs, 730 participants, GRADE: Low) (26, 27). However, Li et al. found that Tai Chi can significantly improve GDS score (MD=-2.81,95%CI [-3.48, -2.14], *P*<0.001, *I* ^2^ = 45%, 2RCTs, 110 participants, GRADE: Very low) (20). The overlap between primary studies in 3 systematic reviews (a total of 5 studies) was 30%.

##### 3.4.2.2 Exercise therapy

Exercise therapy is a planned, structured, repetitive and purposeful form of PA, including various types of joint functional training, plyometric training, and aerobic training (30). Two systematic reviews demonstrated that exercise therapy can improve depressive symptoms significantly compared to non-active control groups (SMD=-0.33, 95% CI [−0.56, −0.10], 8 RCTs, 393 participants, GRADE: Low; SMD=−0.37, 95% CI [-0.64, -0.10], *P*=0.007, *I* ^2^ = 68%, 6 RCTs, 837 participants, GRADE: Low) (9, 21). The overlap between primary studies (a total of 26 studies) was 7.69%.

##### 3.4.2.3 Dance

Dance is a universal cultural expression that transcends time and provides valuable physical activity, extremely advantageous together with music therapy facilitating emotional bonding and enhancing adherence in older adults (31, 32). A meta-analysis by Wu et al. in 2021 found no significant difference in depression scores between the dance group and control group (SMD=−0.37, 95% CI [-1.11, 0.38], *P*=0.34, *I* ^2^ = 80%, 3 RCTs, 157 participants, GRADE: Low) (25).

#### 3.4.3 Psychosocial intervention

Psychosocial intervention refers to planned step-by-step processes aimed at influencing the psychological activities, personality traits, or psychological problems based on psychological theories, with the goal of achieving desired changes (7). Three systematic reviews have examined psychosocial interventions (9, 18, 23), with two focusing specifically on cognitive behavioral therapy (23) and mindfulness-based intervention (18), and one exploring the effect of psychosocial intervention type not specified.

##### 3.4.3.1 Cognitive behavioral therapy

Cognitive behavioral therapy is a psychosocial intervention aimed at improving mental health by developing strategies to change negative thoughts and beliefs, thus adapting to adverse emotions and behaviors (33). Orgeta et al. conducted a systematic review which revealed no significant difference between cognitive behavioral therapy and no treatment (treatment as usual) or non-specific psychosocial activities for MCI with depression (SMD=0.03,95% CI [-0.18, 0.24], *P*=0.77, *I* ^2^ = 0%, 3 RCTs, 339 participants, GRADE: Moderate) (23).

##### 3.4.3.2 Mindfulness-based intervention

Mindfulness-based intervention was originally developed for mindfulness-based intervention stress reduction program, which help individuals improve their overall well-being (34, 35). In 2023, Leow et al. conducted a meta-analysis on MBI for treating depression, and the results favored the control group receiving psychoeducation, health education programs, cognitive training and treatment as usual (SMD=0.29, 95% CI [0.00, 0.57], *P*=0.05, *I* ^2^ = 0%, 4 RCTs, 196 participants, GRADE: Very Low) (18).

##### 3.4.3.3 Psychosocial intervention type not specified

The meta-analysis focusing on psychosocial intervention type not specified revealed no significant difference in depression scores between the psychosocial intervention group and the non-active control group (SMD=-0.13, 95% CI [−0.40, 0.14], 6 RCTs, 277 participants, GRADE: Low) (9).

#### 3.4.4 Music therapy

Music therapy involves various activities designed to improve mood and motivation, whether through listening to selected recordings or live performances by a music therapist, or through actively participating in the creation of music (36, 37). Jordan et al. reported the effects of four types of music therapies (passive, active, reminiscence, and music with movement) on depression in MCI patients through a narrative analysis (17). Lack of quantitative data from this included narrative review, we were unable to present meta-analyses with GRADE assessments of music therapy on depression in MCI patients; instead, a narrative description was provided.

Passive music therapy involves listening to pre-recorded or live music performed by a therapist, that is, music listening. The review showed that passive music therapy can significantly improve depression according to two single-group studies.

Active music therapy refers to actively participating in the creation of music, that is, music playing. Two RCTs on active music therapy yielded contradictory results on depression when assessed by different scales: one study showed a significant reduction of active music therapy than gymnastics in depression when assessed by GDS scores, while another study found no significant difference between active music therapy and no intervention in depression when assessed by BDI scores.

Reminiscence music therapy refers to using music to evoke memories and emotions from the past, that is, music listening purpose to evoke memories. The narrative review did not show significant improvement in depression after reminiscence music therapy.

Music with movement combines music with physical movement or dance. The review showed significant improvement in depression in favor of music with movement.

#### 3.4.5 Health education

Health education involves educational activities and processes that help individuals and groups gain health knowledge, establish health concepts, and adopt beneficial health behaviors through information dissemination and behavioral interventions (38, 39). A meta-analysis found that health education cannot significantly improve depression compared to a non-active control group (SMD=-0.12, 95% CI [−0.44, 0.20], 6 RCTs, 216 participants, GRADE: Low) (9).

#### 3.4.6 Multimodal non-pharmacological interventions

Multimodal non-pharmacologic interventions involve the use of two or more non-pharmacologic therapies. Two systematic reviews have examined multimodal non-pharmacologic interventions (9, 22, 40).

Xu et al. compared an exercise therapy plus cognitive intervention to non-active control, and the meta-analysis showed no significant difference in improving depression between the two groups (MD=-0.33, 95% CI [−0.68, 0.02], 2 RCTs, 173 participants, GRADE: Low) (9).

Mei et al. compared multimodal non-pharmacologic interventions, including cognitive intervention, physical activity, and stress management, to placebo or positive intervention (22). The meta-analysis found a significant improvement in depression score for multimodal non-pharmacologic interventions (2 RCTs, 52 participants, SMD=-0.83,95% CI [-1.41, -0.26], *P*=0.005, *I* ^2^ = 0%, GRADE: Low).

## 4 Discussion

### 4.1 Key findings

Our comprehensive review of 12 systematic reviews, spanning from 2011 to 2023, highlights the diverse landscape of six types of non-pharmacological interventions targeting at depressive symptoms in MCI. These interventions include cognitive interventions (general, computer-based, cognitive rehabilitation/stimulation), physical activity (Tai Chi, exercise therapy, dance), psychosocial interventions (cognitive behavioral therapy, mindfulness-based intervention), music therapy, health education, and multimodal non-pharmacological interventions, each demonstrating varying levels of effectiveness. Specifically, cognitive interventions (general or computer-based) and exercise therapy (a type of physical activity) show preliminary potential to improve depressive symptoms. In contrast, dance (a type of physical activity), health education, and psychosocial interventions do not show significant effects based on the systematic reviews included. The effectiveness of Tai Chi (a type of physical activity), music therapy and multimodal non-pharmacological interventions showed inconsistency across studies. The strength of the evidence is rated as low to very low for all non-pharmacological interventions, except for that on cognitive intervention (general) and cognitive behavior therapy (a psychosocial intervention).

### 4.2 Strengths and weaknesses of the review

This study undertook a comprehensive overview on non-pharmacological interventions for depression in MCI, with no language restrictions. This effort not only responds to the WHO’s call for evidence-based practices in managing depression in non-specialized health settings, but also addresses the critical implications of depression on cognitive health in aging populations. We included systematic reviews of any types of primary studies as well as narrative reviews to obtain this complete evidence map of all potential non-pharmacological interventions at systematic review level. And this study can provide information of inconsistent results from different systematic reviews on the same topic. We assessed the outcomes on depression by the GRADE to determine the information on the strength of evidence. We critically appraised the included systematic reviews by AMSTAR2 tool to provide levels of methodological quality of included evidence. The quality assessments facilitate the readers interpreting the effects of non-pharmacological interventions with reference to the quality of the supporting evidence, and thus making the clinical decisions more rationally. To avoid the potential duplicate use of primary studies results and the ensuing false precision in the analysis, we have calculated the CCA to evaluate the overlap of primary studies across the systematic reviews. The observed low CCA (1.8%) indicates minimal overlap among the original studies, suggesting that our conclusions are based on a diverse set of primary studies. Lastly, this study only included MCI patients considering the heterogeneity among the population of healthy population, MCI, and dementia.

The review has limitations as well. Most of these systematic reviews acknowledged the poor quality of the included primary trials, which is also reflected in GRADE scores. Heterogeneity exists among the included systematic reviews due to variations in diagnostic criteria, study design of included primary studies, definition of interventions, comparison groups, scales to assess depressive symptoms, which complicates data interpretation and affects the reliability and generalizability of our findings. For example, a systematic review exploring the effect of active music therapy revealed inconsistent findings on the improvements of depressive symptoms, that is, being effective as assessed by GDS while ineffective assessed by BDI (17). Inconsistent measurements of depressive symptoms hinder the integration and comparison of the effect of non-pharmacological interventions.

### 4.3 Comparison to current recommendations

From this comprehensive overview of systematic reviews, there is some evidence of improvement in depression by non-pharmacological interventions including cognitive interventions (general or computer-based) and physical activity (exercise therapy). Our review aligns with previous recommendations on cognitive interventions by MCI guidelines and consensus statements. *Petersen* et al. stated that clinicians may recommend cognitive interventions for MCI patients (41); and *Kandiah* et al. recommended that the management strategy should at least include cognitive training (42). The recommendations mainly focus on cognitive training due to its potential in improving the overall cognitive function and functions of multiple cognitive domains (43). Our study provided certain evidence for supporting the use of cognitive interventions, general or computer-based, in improving depressive symptoms of MCI patients; but the importance of dissecting and clarifying the content of cognitive interventions is stressed to further understand the potential varied impacts. Previous guidelines and consensus statements have recommended physical activity in management of MCI: physical activity may be recommended to adults with MCI to reduce the risk of cognitive decline (7); and clinicians should recommend regular exercise (twice/week) as part of an overall approach to management (41). Physical activity is also recommended involving aerobic exercise for dementia from the perspective of improving cognitive outcomes (44). However, the recommendation of physical activity related to improvement of depression remains unclear. Besides exercise therapy, our overview obtained evidence on Tai Chi and Dance in management of depression in MCI, but the conclusions from systematic reviews were inconsistent or negative (20, 25–27). The guideline also mentioned that the current recommendation about physical activity is conditional based on low quality of evidence (7).

Besides the cognitive interventions and physical activity (exercise therapy), a previous analysis of 13 available MCI clinical practice guidelines recommended non-pharmacological interventions such as dietary and nutritional interventions, acupuncture, and counseling (2). In our overview of systematic reviews, there is no evidence found of dietary and nutritional interventions, and acupuncture on depression in MCI. However, dietary interventions and acupuncture have shown potential benefits for cognitive function in MCI population (45, 46). *Xu* et al. revealed no significant effect of health education on depression in MCI (9). However, *Petersen* et al. recommended that clinicians should counsel patients and families to discuss long-term planning topics such as advance directives, driving safety, finances, and estate planning (41). Health education and counseling usually come together but perhaps with slight difference. Health education is to equip patients with accurate and right knowledge, while counseling helps them to apply that knowledge by changing their attitudes and behaviors.

Despite the findings in this overview indicating that psychosocial interventions, such as cognitive behavioral therapy and mindfulness-based interventions, did not result in significant improvements in depressive symptoms in MCI patients, this does not negate their potential values. According to the WHO mhGAP Intervention Guide, psychosocial treatments recommended for adults with moderate to severe depressive disorder include psychoeducation, addressing psychosocial stressors, reactivating social networks, brief psychological treatments, and offering regular follow-up (47). While the WHO also notes the current lack of sufficient evidence for these interventions in managing depression and reducing the risk of cognitive decline/dementia, yet emphasizes their importance for other benefits without advising against their use (7). Psychosocial interventions may still be considered valuable in therapeutic strategies for MCI patients, potentially due to offering benefits beyond direct symptom relief such as enhancing overall well-being and quality of life.

### 4.4 Relevance to clinical practice and research

The analyses in our overview mostly obtain the general effect of non-pharmacological interventions on depressive outcomes of MCI, however, clinical decisions often depend on individual patient characteristics. In need of individually based clinical practice guidelines, it is necessary to report analyses on specific MCI subgroups by identifying whether there is consistency of the effect of non-pharmacological interventions among different patient groups. In view of its value to both patients and clinicians, we planned to perform subgroup analyses regarding different clinical types of MCI, such as amnestic versus non-amnestic MCI, MCI due to Alzheimer’s disease versus due to other conditions. However, only one included SR explicitly addressed patients with amnestic MCI while the others did not specify the type (24). We were unable to conclude on the effect of non-pharmacological interventions regarding different types of MCI currently. As no specific accepted test and cutoff score by the guidelines for the diagnosis of MCI (2), researches on the difference between different types of MCI mostly focus on how to differentiate one from another in diagnosis (48), but few discussed their potential varied responses to certain interventions. For the effectiveness evaluation studies in future, it is advocated to design more specifically on population of MCI, thereby drawing clearer pictures of the overall treatment effect of non-pharmacological interventions across certain MCI subgroups, and perhaps providing some patients with its benefits and protecting others from its harm. Moreover, researches comparing the effect of certain non-pharmacological intervention between different schemes (e.g. long-term versus short-term, high- versus low-frequency) are also expected to find the ‘optimal component’ of this intervention in playing the acts on depressive symptoms of MCI population.

Cognitive decline or dementia is a rapidly growing global public health issue. Except for age, several potentially modifiable risk factors and medical conditions are associated with the increased risk, meaning that the prevention of cognitive decline is possible through a public health approach, including the implementation of key interventions target at different risk factors and comorbidities (7). Non-pharmacological interventions have shown potential benefits for medical conditions related to cognitive decline (e.g. diabetes, hypertension) as well as dietary risk factors (e.g. smoking cessation) of MCI (49–51). Even though the implementation of non-pharmacological interventions in MCI patients for managing depression needs more robust evidence, clinicians may recommend these interventions from the perspective of reducing risk factors of cognitive decline and commodities. The patients with MCI could at least consider the convenient non-pharmacological interventions such as changing physical inactivity and unhealthy diets.

## 5 Conclusion

This overview highlights the diverse landscape of non-pharmacological interventions, including cognitive interventions, physical activity, psychosocial interventions, music therapy, health education, and multimodal non-pharmacological interventions, for depressive symptoms in MCI. Cognitive interventions (general or computer-based) and exercise therapy (a type of physical activity) show preliminary potential to improve depressive symptoms, while others do not show significant effects or relate to confused effects. Further methodologically rigorous and adequately powered primary studies are necessary for each of these non-pharmacological interventions, with reporting on the components of the interventions clearly in MCI patients.

## Data Availability

The original contributions presented in the study are included in the article/**Supplementary Material**. Further inquiries can be directed to the corresponding author/s.
