# Supplementary material for: Effects of non-pharmacological interventions on the depressive outcomes in people with mild cognitive impairment: an overview of systematic reviews
Source: Front Psychiatry. 2025 Jan 15;15:1415113. doi: 10.3389/fpsyt.2024.1415113 (PMC11775744; doi:10.3389/fpsyt.2024.1415113)
Supplement: Supplementary file 1 [file Table1.docx]

**Supplementary files**

**Supplementary Table 1**

**Search strategy**

| **Data Sources** | **Search Terms** |
| --- | --- |
| **Pubmed** | (((((("Depressive Disorder"[Mesh]) OR (((((((((((((((Depressive Disorder*[Title/Abstract]) OR (Disorder*, Depressive[Title/Abstract])) OR (Neurosis, Depressive[Title/Abstract])) OR (Depressive Neurose*[Title/Abstract])) OR (Depressive Neurosis[Title/Abstract])) OR (Neurose*, Depressive[Title/Abstract])) OR (Depression*, Endogenous[Title/Abstract])) OR (Endogenous Depression*[Title/Abstract])) OR (Depressive Syndrome*[Title/Abstract])) OR (Syndrome*, Depressive[Title/Abstract])) OR (Depression*, Neurotic[Title/Abstract])) OR (Neurotic Depression*[Title/Abstract])) OR (Melancholia*[Title/Abstract])) OR (Unipolar Depression*[Title/Abstract])) OR (Depression*, Unipolar[Title/Abstract]))) OR ("depression"[Mesh])) OR ((((((Depression[Title/Abstract]) OR (depress*[Title/Abstract])) OR (Depressive Symptom*[Title/Abstract])) OR (Symptom*, Depressive[Title/Abstract])) OR (Emotional Depression[Title/Abstract])) OR (Depression, Emotional[Title/Abstract])))) AND ((((((systematic review*[Title/Abstract]) OR (meta-analy*[Title/Abstract])) OR (meta analy*[Title/Abstract])) OR ("Meta-Analysis" [Publication Type])) OR ("Systematic Review" [Publication Type])))) AND  ((cognitive dysfunction[Title/Abstract]) OR (mild cognitive impairment*[Title/Abstract]) OR (MCI[Title/Abstract]) OR (cognitive disorder[Title/Abstract]) OR (cognitive impairment[Title/Abstract]) OR (neurocognitive disorder[Title/Abstract]) OR (cognitive decline[Title/Abstract]) OR (mental deterioration*[Title/Abstract]) OR ("Cognitive Dysfunction"[Mesh])) |
| **Wanfang** | (((主题:(认知) or 题名或关键词:(认知)) and (主题:(损伤) or 题名或关键词:(损伤) or 主题:(障碍) or 题名或关键词:(障碍) or 主题:(减退) or 题名或关键词:(减退) or 主题:(损害) or 题名或关键词:(损害))) or 主题:(MCI) or 题名或关键词:(MCI)) and (主题:(抑郁) or 题名或关键词:(抑郁) or 主题:(忧郁) or 题名或关键词:(忧郁)) and (主题:(系统评价) or 题名或关键词:(系统评价) or 主题:(meta) or 题名或关键词:(meta) or 主题:(荟萃分析) or 题名或关键词:(荟萃分析) or 主题:(元分析) or 题名或关键词:(元分析) ) |

**Supplementary Table 2**

**List of full-texts excluded**

| **No.** | **Citations** | **Reasons for exclusion** |
| --- | --- | --- |
| 1 | Oba H, Kobayashi R, Kawakatsu S, Suzuki K, Otani K, Ihara K. Non-pharmacological Approaches to Apathy and Depression: A Scoping Review of Mild Cognitive Impairment and Dementia. Front Psychol. 2022;13:815913. | Not a systematic review |
| 2 | D'Cunha NM, Nguyen D, Naumovski N, McKune AJ, Kellett J, Georgousopoulou EN, et al. A mini-review of virtual reality-based interventions to promote well-being for people living with dementia and mild cognitive impairment. Gerontology. 2019;65(4):430-40. | Not a systematic review |
| 3 | Li JM, Li XF. Effects of mindful ness intervention on mind cognitive impairment: a meta-analysis. Chinese Journal of Alzheimer's Disease and Related Disorders. 2022;5(2):154-61. | No clearly statement of depressive outcome measurement |
| 4 | Inagawa T, Narita Z, Sugawara N, Maruo K, Stickley A, Yokoi Y, et al. A Meta-Analysis of the Effect of Multisession Transcranial Direct Current Stimulation on Cognition in Dementia and Mild Cognitive Impairment. Clinical EEG and Neuroscience. 2019;50(4):273-82. | No clearly statement of depressive outcome measurement |
| 5 | De Siqueira ASS, Yokomizo JE, Jacob-Filho W, Yassuda MS, Aprahamian I. Review of Decision-Making in Game Tasks in Elderly Participants with Alzheimer Disease and Mild Cognitive Impairment. Dementia and Geriatric Cognitive Disorders. 2017;43(1-2):81-8. | No clearly statement of depressive outcome measurement |
| 6 | Loprinzi PD, Blough J, Ryu S, Kang M. Experimental effects of exercise on memory function among mild cognitive impairment: systematic review and meta-analysis. Physician and Sportsmedicine. 2019;47(1):21-6. | No clearly statement of depressive outcome measurement |
| 7 | Johnson M, Lin F. Communication difficulty and relevant interventions in mild cognitive impairment: Implications for neuroplasticity. Topics in Geriatric Rehabilitation. 2014;30(1):18-34. | No clearly statement of depressive outcome measurement |
| 8 | Ahn J, Kim M. Effects of exercise therapy on global cognitive function and, depression in older adults with mild cognitive impairment: A systematic review and meta-analysis. Arch Gerontol Geriatr. 2023;106:104855. | No clearly statement of depressive outcome measurement |
| 9 | Wang XM, Zhang YY, Yu H, Yang FJ, LI YJ, LI Y. Effect of computerized cognitive training in elderly with mild cognitive impairment: a meta-analysis. ClinicalFocus. 2019;34(9). | Not only MCI |
| 10 | Chen YJ, Du SZ, Ding H, Li WT, Xu GH. Effects of Cognitive Therapy Combined with Physical Exercise on the Elderly with Alzheimer's Disease and Mild Cognitive Impairment:a Meta-analysis.Chinese General Practice. 2018;21(32). | Not only MCI |
| 11 | Teselink J, Bawa KK, Koo GK, Sankhe K, Liu CS, Rapoport M, et al. Efficacy of non-invasive brain stimulation on global cognition and neuropsychiatric symptoms in Alzheimer's disease and mild cognitive impairment: A meta-analysis and systematic review. Ageing Research Reviews. 2021;72. | Not only MCI |
| 12 | Wang X, Wang H, Ye Z, Ding G, Li F, Ma J, et al. The neurocognitive and BDNF changes of multicomponent exercise for community-dwelling older adults with mild cognitive impairment or dementia: a systematic review and meta-analysis. Aging (Albany NY). 2020;12(6):4907-17. | Not only MCI |
| 13 | Orgeta V, Qazi A, Spector A, Orrell M. Psychological treatments for depression and anxiety in dementia and mild cognitive impairment: Systematic review and meta-analysis. The British Journal of Psychiatry. 2015;207(4):293-8. | Not only MCI |
| 14 | Leung P, Orrell M, Orgeta V. Social support group interventions in people with dementia and mild cognitive impairment: A systematic review of the literature. International Journal of Geriatric Psychiatry. 2015;30(1):1-9. | Not only MCI |
| 15 | Rios Rincon AM, Miguel Cruz A, Daum C, Neubauer N, Comeau A, Liu L. Digital Storytelling in Older Adults With Typical Aging, and With Mild Cognitive Impairment or Dementia: A Systematic Literature Review. Journal of Applied Gerontology. 2022;41(3):867-80. | Not only MCI |
| 16 | Nagaoka M, Hashimoto Z, Takeuchi H, Sado M. Effectiveness of mindfulness-based interventions for people with dementia and mild cognitive impairment: A meta-analysis and implications for future research. PLoS ONE. 2021;16(8). | Not only MCI |
| 17 | Hu M, Zhang P, Leng M, Li C, Chen L. Animal-assisted intervention for individuals with cognitive impairment: A meta-analysis of randomized controlled trials and quasi-randomized controlled trials. Psychiatry Research. 2018;260:418-27. | Not only MCI |
| 18 | Bird RJ, Hoggard N, Aceves-Martins M. The effect of grape interventions on cognitive and mental performance in healthy participants and those with mild cognitive impairment: a systematic review of randomized controlled trials. Nutr Rev. 2022;80(3):367-380. | Not only MCI |
| 19 | Domenicucci R, Ferrandes F, Sarlo M, Borella E, Belacchi C. Efficacy of ICT-based interventions in improving psychological outcomes among older adults with MCI and dementia: A systematic review and meta-analysis. Ageing Res Rev. 2022;82:101781. | Not only MCI |
| 20 | Han A. Mindfulness-Based Interventions for Older Adults with Dementia or Mild Cognitive Impairment: A Meta-Analysis. Clin Gerontol. 2022;45(4):763-776. | Not only MCI |
| 21 | Chae HJ, Lee SH. Effectiveness of online-based cognitive intervention in community-dwelling older adults with cognitive dysfunction: A systematic review and meta-analysis. Int J Geriatr Psychiatry. 2023;38(1):e5853 | Not only MCI |
| 22 | Yang L, Zhao F, Sun Y, Wang Z, Li Q, Wang H, Lu Y. N-3 Polyunsaturated Fatty Acids in Elderly with Mild Cognitive Impairment: A Systemic Review and Meta-Analysis. J Alzheimers Dis. 2024;99(s1):S81-S95. | Combined with other therapies |
| 23 | Zhang J, Liu J, Li J, Zhang C, Qu M. Non-invasive brain stimulation for improving cognitive function in people with dementia and mild cognitive impairment. Cochrane Database of Systematic Reviews. 2018;2018(7). | Protocol |
| 24 | Orgeta V, Qazi A, Spector AE, Orrell M. Psychological treatments for depression and anxiety in dementia and mild cognitive impairment. Cochrane Database Syst Rev. 2014;2014(1):Cd009125. | Having the updated version |

**Supplementary Table 3**

**The overlap result of primary studies**

| **Primary studies** | **Systematic reviews included** | | | | | | | | | | | |
| --- | --- | --- | --- | --- | --- | --- | --- | --- | --- | --- | --- | --- |
|  | Li 2011(Li et al., 2011) | Simon 2012(Simon et al., 2012) | Mei 2019(Mei et al., 2019) | Zhang 2019(Zhang et al., 2019) | Zhang 2020(Zhang et al., 2020) | Wu 2021(Wu et al., 2021) | Xu 2021(Xu et al., 2021) | Li 2021(Li et al., 2021) | Orgeta 2022(Orgeta et al., 2022) | Jordan 2022(Jordan et al., 2022) | Leow 2023(Leow et al., 2023) | Liu 2023(Liu et al., 2023) |
| Clare 2009(Clare et al., 2009) | √ |  |  |  |  |  |  |  |  |  |  |  |
| Cipriani 2006(Cipriani et al., 2006) | √ |  |  |  |  |  |  |  |  |  |  |  |
| Kurz 2009(Kurz et al., 2009) | √ |  | √ |  |  |  |  |  |  |  |  |  |
| Londos 2008(Londos et al., 2008) | √ |  |  |  |  |  |  |  |  |  |  |  |
| Ng 2006(Ng et al., 2006) | √ |  |  |  |  |  |  |  |  |  |  |  |
| Olazarán 2004(Olazarán et al., 2004) | √ |  |  |  |  |  |  |  |  |  |  |  |
| Rozzini 2007(Rozzini et al., 2007) | √ | √ |  |  |  |  |  |  |  |  |  |  |
| Talassi 2007(Talassi et al., 2007) | √ |  |  |  |  |  |  |  |  |  |  |  |
| Wenisch 2007(Wenisch et al., 2007) | √ |  |  |  |  |  |  |  |  |  |  |  |
| Buschert 2011(Buschert et al., 2011) |  |  | √ |  |  |  |  |  |  |  |  |  |
| Lam 2010(Lam et al., 2011) |  |  |  | √ | √ |  |  |  |  |  |  | √ |
| Lam 2014(Lam et al., 2014) |  |  |  | √ | √ |  |  |  |  |  |  |  |
| Bae 2019(Bae et al., 2019) |  |  |  |  |  |  | √ |  |  |  |  |  |
| Dechamps 2009(Deschamps et al., 2009) |  |  |  |  | √ |  |  | √ |  |  |  |  |
| Dechamps 2010(Dechamps et al., 2010) |  |  |  |  | √ |  |  |  |  |  |  |  |
| Bisbe, 2020(Bisbe et al., 2020) |  |  |  |  |  | √ |  |  |  |  |  |  |
| Wang, 2019(Wang et al., 2020b) |  |  |  |  |  | √ |  |  |  |  |  |  |
| Zhu, 2018(Zhu et al., 2018) |  |  |  |  |  | √ | √ |  |  |  |  | √ |
| Ciarmiello, 2015(Ciarmiello et al., 2015) |  |  |  |  |  |  | √ |  |  |  |  |  |
| Eyre, 2016(Eyre et al., 2016) |  |  |  |  |  |  | √ |  |  |  |  |  |
| Giuli, 2016(Giuli et al., 2016) |  |  |  |  |  |  | √ |  |  |  |  |  |
| Jeong, 2016(Jeong et al., 2016) |  |  |  |  |  |  | √ |  |  |  |  |  |
| Eyre, 2017(Eyre et al., 2017) |  |  |  |  |  |  | √ |  |  |  |  |  |
| Lazarou, 2017(Lazarou et al., 2017) |  |  |  |  |  |  | √ |  |  |  |  |  |
| Belleville, 2018(Belleville et al., 2018) |  |  |  |  |  |  | √ |  | √ |  |  |  |
| Mahendran, 2018(Mahendran et al., 2018) |  |  |  |  |  |  | √ |  |  |  |  |  |
| Klainin-Yobas, 2019(Klainin-Yobas et al., 2019) |  |  |  |  |  |  | √ |  |  |  | √ |  |
| Langoni, 2019(Langoni et al., 2019) |  |  |  |  |  |  | √ |  |  |  |  | √ |
| Larouche, 2018(Larouche et al., 2019) |  |  |  |  |  |  | √ |  |  |  | √ |  |
| Park, 2019(Park et al., 2019a) |  |  |  |  |  |  | √ |  |  |  |  |  |
| Park, 2019(Park et al., 2019b) |  |  |  |  |  |  | √ |  |  |  |  |  |
| Song, 2019(Song and Yu, 2019) |  |  |  |  |  |  | √ |  |  |  |  |  |
| Young, 2019(Young et al., 2019) |  |  |  |  |  |  | √ |  |  |  |  |  |
| Masika, 2020(Masika et al., 2021) |  |  |  |  |  |  | √ |  |  |  |  |  |
| Park, 2020(Park et al., 2020) |  |  |  |  |  |  | √ |  |  |  |  |  |
| Lam, 2020(Lam et al., 2015) |  |  |  |  |  |  | √ |  |  |  |  |  |
| Vidovich, 2015(Vidovich et al., 2015) |  |  |  |  |  |  | √ |  |  |  |  |  |
| Emsaki G, 2017(Emsaki et al., 2017) |  |  |  |  |  |  | √ |  |  |  |  |  |
| Huang, 2019(Huang et al., 2019) |  |  |  |  |  |  |  | √ |  |  |  |  |
| Doshi, 2021(Doshi et al., 2021) |  |  |  |  |  |  |  |  |  |  | √ |  |
| Marcinlak, 2020(Marciniak et al., 2020) |  |  |  |  |  |  |  |  |  |  | √ |  |
| Lu, 2016(Lu et al., 2016) |  |  |  |  |  |  |  |  | √ |  |  |  |
| Rovner, 2018(Rovner et al., 2018) |  |  |  |  |  |  |  |  | √ |  |  |  |
| Cross, 2012(Cross et al., 2012) |  |  |  |  |  |  |  |  |  | √ |  |  |
| Dominguez-Chavez, 2019(Domínguez-Chávez et al., 2019) |  |  |  |  |  |  |  |  |  | √ |  |  |
| Mahendran, 2018* |  |  |  |  |  |  |  |  |  | √ |  |  |
| Biassuti, 2019(Biasutti and Mangiacotti, 2021) |  |  |  |  |  |  |  |  |  | √ |  |  |
| Han,2020(Han et al., 2020) |  |  |  |  |  |  |  |  |  | √ |  |  |
| Wang, 2020(Wang et al., 2020a) |  |  |  |  |  |  |  |  |  |  |  | √ |
| Dan, 2019* |  |  |  |  |  |  |  |  |  |  |  | √ |
| Chang, 2019(Chang et al., 2021) |  |  |  |  |  |  |  |  |  |  |  | √ |

*Note：*Blue represented that this primary study was included in more than one systematic reviews; * : The details of the article were not reported.

**Supplementary Table 4**

**The methodological quality of included systematic reviews assessed by AMSTAR 2**

| **Study/Year** | **Item 1** | **Item 2** | **Item 3** | **Item 4** | **Item 5** | **Item 6** | **Item 7** | **Item 8** | **Item 9** | **Item 10** | **Item 11** | **Item 12** | **Item 13** | **Item 14** | **Item 15** | **Item 16** | **Rating overall confidence in the results of the review** |
| --- | --- | --- | --- | --- | --- | --- | --- | --- | --- | --- | --- | --- | --- | --- | --- | --- | --- |
| Zhang 2020(Zhang et al., 2020) | Y | N | N | PY | Y | Y | N | Y | Y | N | Y | N | Y | N | N | N | Critically low |
| Zhang 2019(Zhang et al., 2019) | Y | N | N | PY | Y | Y | N | Y | Y | N | Y | N | Y | Y | N | N | Critically low |
| Xu 2021(Xu et al., 2021) | Y | N | N | PY | Y | Y | N | Y | Y | N | Y | N | Y | Y | Y | Y | Critically low |
| Wu 2021(Wu et al., 2021) | Y | Y | N | Y | Y | Y | N | Y | Y | N | Y | N | Y | Y | N | Y | Critically low |
|  |  |  |  |  |  |  |  |  |  |  |  |  |  |  |  |  |  |
|  |  |  |  |  |  |  |  |  |  |  |  |  |  |  |  |  |  |
| simon 2012(Simon et al., 2012) | Y | N | N | PY | N | N | N | Y | N | N | No meta-analysis conducted | No meta-analysis conducted | N | Y | No meta-analysis conducted | N | Critically low |
| Orgeta 2022(Orgeta et al., 2022) | Y | Y | N | Y | Y | Y | Y | Y | Y | Y | Y | N | Y | Y | Y | Y | Moderate |
| Mei 2019(Mei et al., 2019) | Y | N | N | PY | Y | Y | N | Y | Y | N | Y | N | Y | N | N | Y | Critically low |
| Liu 2023(Liu et al., 2023) | Y | Y | N | PY | Y | Y | N | Y | Y | N | Y | N | Y | Y | Y | Y | Critically low |
| Li 2021(Li et al., 2021) | Y | N | N | PY | Y | Y | N | Y | Y | N | Y | N | N | N | N | Y | Critically low |
| Li 2011(Li et al., 2011) | Y | N | N | PY | Y | Y | N | Y | N | N | Y | N | N | Y | Y | Y | Critically low |
| Leow 2023(Leow et al., 2023) | Y | Y | N | Y | Y | Y | N | Y | Y | N | Y | N | Y | Y | N | Y | Critically low |
| Jordan 2022(Jordan et al., 2022) | Y | Y | N | PY | Y | Y | N | Y | Y | N | No meta-analysis conducted | No meta-analysis conducted | Y | N | No meta-analysis conducted | Y | Critically low |

Abbreviation: Y-Yes; N-No; PY- Partial Yes

Item 1: Did the research questions and inclusion criteria for the review include the components of PICO;

Item 2: Did the report of the review contain an explicit statement that the review methods were established prior to the conduct of the review and did the report justify any significant deviations from the protocol;

Item 3: Did the review authors explain their selection of the study designs for inclusion in the review;

Item 4: Did the review authors use a comprehensive literature search strategy;

Item 5: Did the review authors perform study selection in duplicate;

Item 6: Did the review authors perform data extraction in duplicate;

Item 7: Did the review authors provide a list of excluded studies and justify the exclusions;

Item 8: Did the review authors describe the included studies in adequate detail;

Item 9: Did the review authors use a satisfactory technique for assessing the risk of bias (RoB) in individual studies that were included in the review;

Item 10: Did the review authors report on the sources of funding for the studies included in the review;

Item 11: If meta-analysis was performed, did the review authors use appropriate methods for statistical combination of results;

Item 12: If meta-analysis was performed, did the review authors assess the potential impact of RoB in individual studies on the results of the meta-analysis or other evidence synthesis;

Item 13: Did the review authors account for RoB in primary studies when interpreting/discussing the results of the review;

Item 14: Did the review authors provide a satisfactory explanation for, and discussion of, any heterogeneity observed in the results of the review;

Item 15: If they performed quantitative synthesis did the review authors carry out an adequate investigation of publication bias (small study bias) and discuss its likely impact on the results of the review;

Item 16: Did the review authors report any potential sources of conflict of interest, including any funding they received for conducting the review?

**Supplementary Table 5**

**The result of GRADE assessment**

| **Quality assessment** | | | | | | | **No of patients** | | **Effect** | | **Quality** | **Importance** |
| --- | --- | --- | --- | --- | --- | --- | --- | --- | --- | --- | --- | --- |
|  |  |  |  |  |  |  |  |  |  |  |  |  |
| **No of studies** | **Design** | **Risk of bias** | **Inconsistency** | **Indirectness** | **Imprecision** | **Other considerations** | **NPT** | **Control** | **Relative (95% CI)** | **Absolute** |  |  |
| **Zhang 2020 (Zhang et al., 2020)-GDS (Better indicated by lower values)** | | | | | | | | | | | | |
| 2 | randomised trials | very serious^1^ | no serious | no serious | serious^2^ | none | 66 | 70 | - | SMD 0.02 lower (0.36 lower to 0.31 higher) | ÅOOO VERY LOW |  |
| **Zhang 2020 (Zhang et al., 2020)-CSD (Better indicated by lower values)** | | | | | | | | | | | | |
| 2 | randomised trials | serious^1^ | no serious | no serious | serious^2^ | none | 231 | 363 | - | SMD 0.01 higher (0.15 lower to 0.18 higher) | ÅÅOO LOW |  |
| **Zhang 2019 (Zhang et al., 2019)-CSD (Better indicated by lower values)** | | | | | | | | | | | | |
| 2 | randomised trials | serious^1^ | no serious | no serious | serious^2^ | none | 231 | 363 | - | MD 0.02 higher (0.23 lower to 0.27 higher) | ÅÅOO LOW |  |
|  | | | | | | | | | | | | |
|  |  |  |  |  |  |  |  |  |  |  |  |  |
| **Orgeta 2022(Orgeta et al., 2022)-GDS-15 (Better indicated by lower values)** | | | | | | | | | | | | |
| 3 | randomised trials | no serious | no serious | no serious | serious^2^ | none | 171 | 168 | - | SMD 0.03 higher (0.18 lower to 0.24 higher) | ÅÅÅO MODERATE |  |
| **Liu 2023 (Liu et al., 2023)-GDS-15/GDS/CSDD (Better indicated by lower values)** | | | | | | | | | | | | |
| 6 | randomised trials | very serious^1^ | no serious | no serious | no serious | none | 404 | 433 | - | SMD 0.37 lower (0.64 lower to 0.10 lower) | ÅÅOO LOW |  |
| **Li 2021 (Li et al., 2021)-GDS (Better indicated by lower values)** | | | | | | | | | | | | |
| 2 | randomised trials | very serious^1^ | no serious | no serious | serious^2^ | none | 51 | 50 | - | MD 2.81 lower (3.71 lower to 1.71 higher) | ÅOOO VERY LOW |  |
| **Leow 2023 (Leow et al., 2023)-GDS (Better indicated by lower values)** | | | | | | | | | | | | |
| 5 | randomised trials | very serious^1^ | no serious | no serious | serious^2^ | none | 95 | 101 | - | SMD 0.29 higher (0.00 higher to 0.57 higher) | ÅOOO VERY LOW |  |
| **Xu 2021 (Xu et al., 2021)-Health education** | | | | | | | | | | | | |
| 6 | randomised trials | serious^1^ | no serious | no serious | serious^2^ | none | 216 | - | - | SMD 0.12 lower (0.44 lower to 0.20 higher) | ÅÅOO LOW |  |
| **Xu 2021 (Xu et al., 2021)-Psychosocial intervention** | | | | | | | | | | | | |
| 6 | randomised trials | serious^1^ | no serious | no serious | serious^2^ | none | 277 | - | - | SMD 0.13 lower (0.40 lower to 0.14 higher) | ÅÅOO LOW |  |
| **Xu 2021 (Xu et al., 2021)-Cognition-based intervention** | | | | | | | | | | | | |
| 15 | randomised trials | serious^1^ | no serious | no serious | no serious | none | 662 | - | - | SMD 0.25 lower (0.46 lower to 0.04 lower) | ÅÅÅO MODERATE |  |
| **Xu 2021(Xu et al., 2021)-Physical exercise** | | | | | | | | | | | | |
| 8 | randomised trials | serious^1^ | no serious | no serious | serious^2^ | none | 393 | - | - | SMD 0.33 lower (0.56 lower to 0.10 lower) | ÅÅOO LOW |  |
| **Xu 2021(Xu et al., 2021)-Physical exercise combined with cognition-based intervention** | | | | | | | | | | | | |
| 2 | randomised trials | serious^1^ | no serious | no serious | serious^2^ | none | 173 | - | - | SMD 0.33 lower (0.68 lower to 0.02 higher) | ÅÅOO LOW |  |
| **Wu 2021(Wu et al., 2021)-GDS/HADS-D** | | | | | | | | | | | | |
| 3 | randomised trials | no serious | serious^3^ | no serious | serious^2^ | none | 79 | 78 | - | SMD 0.37 lower (1.11 lower to 0.38 higher) | ÅÅOO LOW |  |
| **Mei 2019(Mei et al., 2019)-MADR/BDI** | | | | | | | | | | | | |
| 2 | randomised trials | serious^1^ | no serious | no serious | serious^2^ | none | 30 | 30 | - | SMD 0.83 lower (1.41 lower to 0.26 lower) | ÅÅOO LOW |  |

**Supplementary Table 6**

**The abstracts of the included non-English papers**

| **Studies** | **Abstracts** | **Contact Info** |
| --- | --- | --- |
| **Li 2021** | This paper systematically evaluated the effectiveness of Tai Chi on cognitive function in patients with mild cognitive impairment (MCI), providing reference for future clinical practice and research. Databases including CNKI, Wanfang, VIP, PubMed (including Medline database), Web of science, Cochrane library and others were systematically searched for randomized controlled trials exploring the effect of Tai Chi on cognitive function of patients with MCI. Studies were selected, and data were extracted; RevMan5.3 was used to perform analyses. The results showed that GDS (MD = -2.81, 95%CZ [-3.48, -2.14], P = 0.18) and LM-delayed recall scores in the Tai Chi group were superior to the control group, while there were no significant difference in DSB (MD = 0.17, 95%CI [-0.03, 0.36], P = 0.30), DSF (MD = 1.22, 95%CI [-0.68, 3.12], P = 0.21) and MoCA (MD = -1.58, 95%CI [-9.79, 6.64], P = 0.71) between the groups. Therefore, Tai Chi may have a certain positive effect on the cognitive function of patients with MCI, and the research on the rehabilitation effect should be intensified. | **FAN Tonggang**  TEL: 021-65507231  Email: fantonggang@126.com |
| **Mei 2019** | Objectives To systematically review the efficacy of multimodal nonpharmacological interventions in mild cognitive impairment (MCI). Methods An electronically search was conducted in PubMed, EMbase, The Cochrane Library, PsycINFO, Web of Science, CINAHL, VIP, CBM, WanFang Data and CNKI databases from inception to November 2017 to collect randomized controlled trials (RCTs) on multimodal nonpharmacological interventions for MCI. Two reviewers independently screened literature, extracted data and assessed the risk of bias of included studies. Then, meta-analysis was performed by RevMan 5.3 software. Results A total of 12 RCTs involving 1 359 patients were included. The results of meta-analysis showed that there were no statistical differences between two groups in MMSE scores (SMD=0.33, 95%CI-0.13 to 0.7& P=0.16). However, the MoCA scores (SMD=0.52, 95%CI 0.38 to 0.67, P<0.00001) and ADAS-Cog scores (SMD=1.13, 95%CI 0.75 to 1.51, P<0.00001) in the multimodal nonpharmacological interventions group were better than those in the control group. Additionally, multimodal nonpharmacological interventions produced significant effects on ADL (SMD=-0.64, 95%CI -0.83 to-0.45, P<0.00001), QOL-AD (MD=3.65, 95%CI 1.03 to 6.27, P=0.006) and depression (SMD=-0.83, 95%CI -1.41 to-0.26, P=0.005). There were no statistical differences between two groups on conversion rate to Alzheimer's disease (RR=0.27, 95%CI 0.06 to 1.26, P=0.10). Conclusions The current evidence shows that multimodal nonpharmacological interventions are feasible for patients with MCI as they have positive effects on overall abilities, daily living skills, and quality of life and depression. Nevertheless, due to the limited quantity and quality of included studies, more high-quality studies are required to verify the conclusion. | **CAO Hong**  Email: caohong1968@yeah.net |
| **Zhang 2020** | Objective: To evaluate the effects of Tai Chi on cognitive and psychological outcomes in older adults with Mild Cognitive Impairment (MCI). Methods: A computerized search was performed through PubMed, Embase, CENTRAL, CINAHL, Web of Science, the Chinese Biomedical Database, the China National Knowledge Infrastructure, VIP Journal Integration Platform, and Wanfang Med Online for Randomized Controlled Trials (RCTs) which studied the effects of Tai Chi on older patients with MCI. Meta-analysis was performed by using RevMan 5.3 software. Results: Seven RCTs were included. Comparing to regular exercise or usual care, the memory [M D=0.31, 95%CI (0.13,0.61), P<0.01], visuospatial function [SMD=0.51, 95%CI (0.23, 0.91), P<0.01] were improved in the aged with Tai Chi intervention. Conclusion: Tai Chi is efficacious in improving memory and visuospatial function in the aged with MCI, but cannot significantly improve global cognitive functions, executive function, verbal fluency and depression | **JIN Yi**  Email: jinyi6196@163.com |

Reference

Bae, S., Lee, S., Lee, S., Jung, S., Makino, K., Harada, K., Harada, K., Shinkai, Y., Chiba, I., Shimada, H., 2019. The effect of a multicomponent intervention to promote community activity on cognitive function in older adults with mild cognitive impairment: A randomized controlled trial. Complement Ther Med 42, 164-169.

Belleville, S., Hudon, C., Bier, N., Brodeur, C., Gilbert, B., Grenier, S., Ouellet, M., Viscogliosi, C., Gauthier, S., 2018. MEMO+: Efficacy, Durability and Effect of Cognitive Training and Psychosocial Intervention in Individuals with Mild Cognitive Impairment. J Am Geriatr Soc 66, 655-663.

Biasutti, M., Mangiacotti, A., 2021. Music Training Improves Depressed Mood Symptoms in Elderly People: A Randomized Controlled Trial. Int J Aging Hum Dev 92, 115-133.

Bisbe, M., Fuente-Vidal, A., López, E., Moreno, M., Naya, M., de Benetti, C., Milà, R., Bruna, O., Boada, M., Alegret, M., 2020. Comparative Cognitive Effects of Choreographed Exercise and Multimodal Physical Therapy in Older Adults with Amnestic Mild Cognitive Impairment: Randomized Clinical Trial. J Alzheimers Dis 73, 769-783.

Buschert, V., Friese, U., Teipel, S., Schneider, P., Merensky, W., Rujescu, D., Möller, H., Hampel, H., Buerger, K., 2011. Effects of a newly developed cognitive intervention in amnestic mild cognitive impairment and mild Alzheimer's disease: a pilot study. J Alzheimers Dis 25, 679-694.

Chang, J., Chen, Y., Liu, C., Yong, L., Yang, M., Zhu, W., Wang, J., Yan, J., 2021. Effect of Square Dance Exercise on Older Women With Mild Mental Disorders. Front Psychiatry 12, 699778.

Ciarmiello, A., Gaeta, M., Benso, F., Del Sette, M., 2015. FDG-PET in the Evaluation of Brain Metabolic Changes Induced by Cognitive Stimulation in aMCI Subjects. Curr Radiopharm 8, 69-75.

Cipriani, G., Bianchetti, A., Trabucchi, M., 2006. Outcomes of a computer-based cognitive rehabilitation program on Alzheimer's disease patients compared with those on patients affected by mild cognitive impairment. Arch Gerontol Geriatr 43, 327-335.

Clare, L., van Paasschen, J., Evans, S.J., Parkinson, C., T, W.R., Linden, D.E., 2009. Goal-oriented cognitive rehabilitation for an individual with mild cognitive impairment: behavioural and neuroimaging outcomes Neurocase 15, 318-331.

Cross, K., Flores, R., Butterfield, J., Blackman, M., Lee, S., 2012. The effect of passive listening versus active observation of music and dance performances on memory recognition and mild to moderate depression in cognitively impaired older adults. Psychol Rep 111, 413-423.

Dechamps, A., Diolez, P., Thiaudière, E., Tulon, A., Onifade, C., Vuong, T., Helmer, C., Bourdel-Marchasson, I., 2010. Effects of exercise programs to prevent decline in health-related quality of life in highly deconditioned institutionalized elderly persons: a randomized controlled trial. Arch Intern Med 170, 162-169.

Deschamps, A., Onifade, C., Decamps, A., Bourdel-Marchasson, I., 2009. Health-related quality of life in frail institutionalized elderly: effects of a cognition-action intervention and Tai Chi. J Aging Phys Act 17, 236-248.

Domínguez-Chávez, C., Murrock, C., Guerrero, P., Salazar-González, B., 2019. Music therapy intervention in community-dwelling older adults with mild cognitive impairment: A pilot study. Geriatr Nurs 40, 614-619.

Doshi, K., Henderson, S., Fan, Q., Wong, K., Lim, J., 2021. Mindfulness-Based Training Does Not Improve Neuropsychological Outcomes in Mild Cognitive Impairment More Than Spontaneous Reversion Rates: A Randomized Controlled Trial. J Alzheimers Dis 84, 449-458.

Emsaki, G., NeshatDoost, H., Tavakoli, M., Barekatain, M., 2017. Memory specificity training can improve working and prospective memory in amnestic mild cognitive impairment. Dement Neuropsychol 11, 255-261.

Eyre, H., Acevedo, B., Yang, H., Siddarth, P., Van Dyk, K., Ercoli, L., Leaver, A., Cyr, N., Narr, K., Baune, B., Khalsa, D., Lavretsky, H., 2016. Changes in Neural Connectivity and Memory Following a Yoga Intervention for Older Adults: A Pilot Study. J Alzheimers Dis 52, 673-684.

Eyre, H., Siddarth, P., Acevedo, B., Van Dyk, K., Paholpak, P., Ercoli, L., St Cyr, N., Yang, H., Khalsa, D., Lavretsky, H., 2017. A randomized controlled trial of Kundalini yoga in mild cognitive impairment. Int Psychogeriatr 29, 557-567.

Giuli, C., Papa, R., Lattanzio, F., Postacchini, D., 2016. The Effects of Cognitive Training for Elderly: Results from My Mind Project. Rejuvenation Res 19, 485-494.

Han, E., Park, J., Kim, H., Jo, G., Do, H., Lee, B., 2020. Cognitive Intervention with Musical Stimuli Using Digital Devices on Mild Cognitive Impairment: A Pilot Study. Healthcare (Basel) 8, 45.

Huang, N., Li, W., Rong, X., Champ, M., Wei, L., Li, M., Mu, H., Hu, Y., Ma, Z., Lyu, J., 2019. Effects of a Modified Tai Chi Program on Older People with Mild Dementia: A Randomized Controlled Trial. J Alzheimers Dis 72, 947-956.

Jeong, J., Na, H., Choi, S., Kim, J., Na, D., Seo, S., Chin, J., Park, S., Kim, E., Han, H., Han, S., Yoon, S., Lee, J., Park, K., Moon, S., Park, M., Choi, M., Han, I., Lee, J., Lee, J., Shim, Y., Kim, J., 2016. Group- and Home-Based Cognitive Intervention for Patients with Mild Cognitive Impairment: A Randomized Controlled Trial. Psychother Psychosom, 198-207.

Jordan, C., Lawlor, B., Loughrey, D., 2022. A systematic review of music interventions for the cognitive and behavioural symptoms of mild cognitive impairment (non-dementia). J Psychiatr Res 151, 382-390.

Klainin-Yobas, P., Kowitlawakul, Y., Lopez, V., Tang, C., Hoek, K., Gan, G., Lei, F., Rawtaer, I., Mahendran, R., 2019. The effects of mindfulness and health education programs on the emotional state and cognitive function of elderly individuals with mild cognitive impairment: A randomized controlled trial. J Clin Neurosci 68, 211-217.

Kurz, A., Pohl, C., Ramsenthaler, M., Sorg, C., 2009. Cognitive rehabilitation in patients with mild cognitive impairment. Int J Geriatr Psychiatry 24, 163-168.

Lam, L., Chan, W., Kwok, T., Chiu, H., 2014. Effectiveness of Tai Chi in maintenance of cognitive and functional abilities in mild cognitive impairment: a randomised controlled trial. Hong Kong Med J 20.

Lam, L., Chan, W., Leung, T., Fung, A., Leung, E., 2015. Would older adults with mild cognitive impairment adhere to and benefit from a structured lifestyle activity intervention to enhance cognition?: a cluster randomized controlled trial. PLoS One. PLoS One 10, e0118173.

Lam, L., Chau, R., Wong, B., Fung, A., Lui, V., Tam, C., Leung, G., Kwok, T., Chiu, H., Ng, S., Chan, W., 2011. Interim follow-up of a randomized controlled trial comparing Chinese style mind body (Tai Chi) and stretching exercises on cognitive function in subjects at risk of progressive cognitive decline. Int J Geriatr Psychiatry 26, 733-740.

Langoni, C., Resende, T., Barcellos, A., Cecchele, B., da Rosa, J., Knob, M., Silva, T., Diogo, T., da Silva, I., Schwanke, C., 2019. The effect of group exercises on balance, mobility, and depressive symptoms in older adults with mild cognitive impairment: a randomized controlled trial. Clin Rehabil 33, 439-449.

Larouche, E., Hudon, C., Goulet, S., 2019. Mindfulness mechanisms and psychological effects for aMCI patients: A comparison with psychoeducation. Complement Ther Clin Pract 34, 93-104.

Lazarou, I., Parastatidis, T., Tsolaki, A., Gkioka, M., Karakostas, A., Douka, S., Tsolaki, M., 2017. International Ballroom Dancing Against Neurodegeneration: A Randomized Controlled Trial in Greek Community-Dwelling Elders With Mild Cognitive impairment. Am J Alzheimers Dis Other Demen 32, 489-499.

Leow, Y., Rashid, N.L.B.A., Klainin-Yobas, P., Zhang, Z., Wu, X.V., 2023. Effectiveness of mindfulness‐based interventions on mental, cognitive outcomes and neuroplastic changes in older adults with mild cognitive impairment: A systematic review and meta‐analysis. Journal of Advanced Nursing 79, 4489-4505.

Li, H., Li, J., Li, N., Li, B., Wang, P., Zhou, T., 2011. Cognitive intervention for persons with mild cognitive impairment: A meta-analysis. Ageing Res Rev 10, 285-296.

Li, W., Xiang, Q., Fan, T., 2021. A Meta-analysis of efficacy of Tai Chi on cognitive function in patients with mild cognitive impairment. Clinical Journal of Chinese Medicine 13, 129-136.

Liu, X., Wang, G., Cao, Y., 2023. The effectiveness of exercise on global cognitive function, balance, depression symptoms, and sleep quality in patients with mild cognitive impairment: A systematic review and meta-analysis. Geriatric Nursing 51, 182-193.

Londos, E., Boschian, K., Lindén, A., Persson, C., Minthon, L., Lexell, J., 2008. Effects of a goal-oriented rehabilitation program in mild cognitive impairment: a pilot study. Am J Alzheimers Dis Other Demen 23, 177-183.

Lu , Y., Ellis, J., Yang, Z., Weaver, M., Bakas, T., Austrom, M., Haase, J., 2016. Satisfaction With a Family-Focused Intervention for Mild Cognitive Impairment Dyads. J Nurs Scholarsh 48, 334-344.

Mahendran, R., Gandhi, M., Moorakonda, R., Wong, J., Kanchi, M., Fam, J., Rawtaer, I., Kumar, A., Feng, L., Kua, E., 2018. Art therapy is associated with sustained improvement in cognitive function in the elderly with mild neurocognitive disorder: findings from a pilot randomized controlled trial for art therapy and music reminiscence activity versus usual care. Trials 19, 615.

Marciniak, R., Šumec, R., Vyhnálek, M., Bendíčková, K., Lázničková, P., Forte, G., Jeleník, A., Římalová, V., Frič, J., Hort, J., Sheardová, K., 2020. The Effect of Mindfulness-Based Stress Reduction (MBSR) on Depression, Cognition, and Immunity in Mild Cognitive Impairment: A Pilot Feasibility Study. Clin Interv Aging 15, 1365-1381.

Masika, G., Yu, D., Li, P., 2021. Can Visual Art Therapy Be Implemented With Illiterate Older Adults With Mild Cognitive Impairment? A Pilot Mixed-Method Randomized Controlled Trial. J Geriatr Psychiatry Neurol 34, 76-86.

Mei, X., Zhao, X., Wang, Y., Wang, L., Cao, H., 2019. Efficacy of multimodal nonpharmacological interventions in mild cognitive impairment: A meta-analysis. Chinese Journal of Evidence-Based Medicine 19, 180-188.

Ng, S., Lo, A., Lee, G., Lam, M., Yeong, E., Koo, M., Tsoi, S., Chow, D., Lau, O., Fung, K., Lai, A., Fung, D., Tam, A., So, S., Lau, V., 2006. Report of the outcomes of occupational therapy programmes for elderly persons with mild cognitive impairment (MCI) in community elderly centres. Hong Kong Journal of Occupational Therapy 16, 16-22.

Olazarán, J., Muñiz, R., Reisberg, B., Peña-Casanova, J., del Ser, T., Cruz-Jentoft, A., Serrano, P., Navarro, E., García, d.l.R.M., Frank, A., Galiano, M., Fernández-Bullido, Y., Serra, J., González-Salvador, M., Sevilla, C., 2004. Benefits of cognitive-motor intervention in MCI and mild to moderate Alzheimer disease. Neurology 63, 2348-2353.

Orgeta, V., Leung, P., del-Pino-Casado, R., Qazi, A., Orrell, M., Spector, A.E., Methley, A.M., 2022. Psychological treatments for depression and anxiety in dementia and mild cognitive impairment. Cochrane Database of Systematic Reviews 4, CD009125.

Park, H., Park, J., Na, H., Hiroyuki, S., Kim, G., Jung, M., Kim, W., Park, K., 2019a. Combined Intervention of Physical Activity, Aerobic Exercise, and Cognitive Exercise Intervention to Prevent Cognitive Decline for Patients with Mild Cognitive Impairment: A Randomized Controlled Clinical Study. J Clin Med 8, 940.

Park, J., Kim, S., Kim, E., Lee, B., Jeong, J., Na, H., Choi, S., Kang, D., Park, K., 2019b. Effect of 12-week home-based cognitive training on cognitive function and brain metabolism in patients with amnestic mild cognitive impairment. Clin Interv Aging 14, 1167-1175.

Park, J., Liao, Y., Kim, D., Song, S., Lim, J., Park, H., Lee, Y., Park, K., 2020. Feasibility and Tolerability of a Culture-Based Virtual Reality (VR) Training Program in Patients with Mild Cognitive Impairment: A Randomized Controlled Pilot Study. Int J Environ Res Public Health 17, 3030.

Rovner, B., Casten, R., Hegel, M., Leiby, B., 2018. Preventing Cognitive Decline in Black Individuals With Mild Cognitive Impairment: A Randomized Clinical Trial. JAMA Neurol 75, 1487-1493.

Rozzini, L., Costard, i.D., Chilovi, B., Franzoni, S., Trabucchi, M., Padovani, A., 2007. Efficacy of cognitive rehabilitation in patients with mild cognitive impairment treated with cholinesterase inhibitors. Int J Geriatr Psychiatry 22, 356-360.

Simon, S.S., Yokomizo, J.E., Bottino, C.M.C., 2012. Cognitive intervention in amnestic mild cognitive Impairment: A systematic review. Neuroscience and Biobehavioral Reviews 36, 1163-1178.

Song, D., Yu, D., 2019. Effects of a moderate-intensity aerobic exercise programme on the cognitive function and quality of life of community-dwelling elderly people with mild cognitive impairment: A randomised controlled trial. Int J Nurs Stud 93, 97-105.

Talassi, E., Guerreschi, M., Feriani, M., Fedi, V., Bianchetti, A., Trabucchi, M., 2007. Effectiveness of a cognitive rehabilitation program in mild dementia (MD) and mild cognitive impairment (MCI): a case control study. Arch Gerontol Geriatr 44 Suppl 1, 391-399.

Vidovich, M., Lautenschlager, N., Flicker, L., Clare, L., McCaul, K., Almeida, O., 2015. The PACE study: a randomized clinical trial of cognitive activity strategy training for older people with mild cognitive impairment. Am J Geriatr Psychiatry 23, 360-372.

Wang, L., Wu, B., Tao, H., Chai, N., Zhao, X., Zhen, X., Zhou, X., 2020a. Effects and mediating mechanisms of a structured limbs-exercise program on general cognitive function in older adults with mild cognitive impairment: A randomized controlled trial. Int J Nurs Stud 110, 103706.

Wang, S., Yin, H., Meng, X., Shang, B., Meng, Q., Zheng, L., Wang, L., Chen, L., 2020b. Effects of Chinese square dancing on older adults with mild cognitive impairment. Geriatr Nurs 41, 290-296.

Wang, X., Zhang, Y., Yu, H., Yang, F., Li, Y., Li, Y., 2019. Efect of computerized cognitive training in elderly with mild cognitive impairment: a meta-analysis. Clinical Focus 34, 843-849.

Wenisch, E., Cantegreil-Kallen, I., De Rotrou, J., Garrigue, P., Moulin, F., Batouche, F., Richard, A., De Sant'Anna, M., Rigaud, A., 2007. Cognitive stimulation intervention for elders with mild cognitive impairment compared with normal aged subjects: preliminary results. Aging Clin Exp Res 19, 316-322.

Wu, V.X., Chi, Y., Lee, J.K., Goh, H.S., Chen, D.Y.M., Haugan, G., Chao, F.F.T., Klainin-Yobas, P., 2021. The effect of dance interventions on cognition, neuroplasticity, physical function, depression, and quality of life for older adults with mild cognitive impairment: A systematic review and meta-analysis. International Journal of Nursing Studies 122, 104025.

Xu, Z., Sun, W., Zhang, D., Chung, V.C.-H., Wong, S.Y.-S., 2021. Comparative effectiveness of non-pharmacological interventions for depressive symptoms in mild cognitive impairment: Systematic review with network meta-analysis. Aging & Mental Health 26, 2129-2135.

Young, D.K., Ng, P.Y., Cheng, D., 2019. Psychoeducation Group on Improving Quality of Life of Mild Cognitive Impaired Elderly. Research on Social Work Practice 29, 303-310.

Zhang, Q., Hu, J., Wei, L., Cao, R., Ma, R., Song, H., Jin, Y., 2019. Effects of traditional Chinese exercise on cognitive and psychological outcomes in older adults with mild cognitive impairment: A systematic review and meta-analysis. Medicine (Baltimore) 98, e14581.

Zhang, Q., Song, H., Cao, R., Sun, X., Jin, Y., 2020. Effects of Tai Chi on cognitive function for aged with Mild Cognitive Impairment: a Meta-analysis. Evidence-based Nursing 20, 865-871.

Zhu, Y., Wu, H., Qi, M., Wang, S., Zhang, Q., Zhou, L., Wang, S., Wang, W., Wu, T., Xiao, M., Yang, S., Chen, H., Zhang, L., Zhang, K., Ma, J., Wang, T., 2018. Effects of a specially designed aerobic dance routine on mild cognitive impairment. Clin Interv Aging, 1691-1700.
